# Patient and physician factors associated with Oncotype DX and adjuvant chemotherapy utilization for breast cancer patients in New Hampshire, 2010–2016

**DOI:** 10.1186/s12885-020-07355-6

**Published:** 2020-09-03

**Authors:** Thomas M. Schwedhelm, Judy R. Rees, Tracy Onega, Ronnie J. Zipkin, Andrew Schaefer, Maria O. Celaya, Erika L. Moen

**Affiliations:** 1 Department of Biomedical Data Science, Dartmouth Geisel School of Medicine, Lebanon, NH USA; 2 New Hampshire State Cancer Registry, Lebanon, NH USA; 3 Department of Epidemiology, Dartmouth Geisel School of Medicine, Lebanon, NH USA; 4 The Dartmouth Institute for Health Policy and Clinical Practice, Lebanon, NH USA

**Keywords:** Oncotype DX, Breast cancer, Adjuvant chemotherapy

## Abstract

**Background:**

Oncotype DX® (ODX) is used to assess risk of disease recurrence in hormone receptor positive, HER2-negative breast cancer and to guide decisions regarding adjuvant chemotherapy. Little is known about how physician factors impact treatment decisions. The purpose of this study was to examine patient and physician factors associated with ODX testing and adjuvant chemotherapy for breast cancer patients in New Hampshire.

**Methods:**

We examined New Hampshire State Cancer Registry data on 5630 female breast cancer patients diagnosed from 2010 to 2016. We performed unadjusted and adjusted hierarchical logistic regression to identify factors associated with a patient’s receipt of ODX, being recommended and receiving chemotherapy, and refusing chemotherapy. We calculated intraclass correlation coefficients (ICCs) to examine the proportion of variance in clinical decisions explained by between-physician and between-hospital variation.

**Results:**

Over the study period, 1512 breast cancer patients received ODX. After adjustment for patient and tumor characteristics, we found that patients seen by a male medical oncologist were less likely to be recommended chemotherapy following ODX (OR = 0.50 (95% CI = 0.34–0.74), *p* < 0.01). Medical oncologists with more clinical experience (reference: less than 10 years) were more likely to recommend chemotherapy (20–29 years: OR = 4.05 (95% CI = 1.57–10.43), *p* < 0.01; > 29 years: OR = 4.48 (95% CI = 1.68–11.95), *p* < 0.01). A substantial amount of the variation in receiving chemotherapy was due to variation between physicians, particularly among low risk patients (ICC = 0.33).

**Conclusions:**

In addition to patient clinicopathologic characteristics, physician gender and clinical experience were associated with chemotherapy treatment following ODX testing. The significant variation between physicians indicates the potential for interventions to reduce variation in care.

## Background

Breast cancer (BC) is the leading cause of cancer in women worldwide and is the second leading cause of cancer death in women [1]. Hormone receptor (HR) positive (defined as estrogen receptor and/or progesterone receptor positive), axillary lymph node (LN) negative BC is the most common subtype in the United States [2]. The treatment paradigm has shifted in the past decade for BC, especially for this subtype [3–5]. Adjuvant chemotherapy had previously been recommended for all BC patients and resulted in improved mortality rates [6, 7]. However, risk stratification of women with HR positive, LN negative BC is a priority, because about 85% of these women are at low risk of disease recurrence with endocrine-modulating therapy alone and thus are unlikely to benefit from adjuvant chemotherapy [8, 9].

Currently, there exist multiple methods to predict risk of 10-year disease recurrence and the potential benefit of chemotherapy [10–12]. Oncotype DX® (Genomic Health Inc., Redwood City, CA) (ODX) is a widely-used prognostic breast cancer test which analyzes gene expression of 16 tumor-specific genes and 5 reference genes [11, 13]. It was commercially introduced in the United States in 2004 and shortly thereafter was recommended in guidelines released by the American Society for Clinical Oncology (ASCO) and the National Comprehensive Cancer Network (NCCN) [14, 15]. The assay provides an integer Recurrence Score (RS), ranging from 0 to 100, indicating low risk (RS < 18), intermediate risk (RS 18–30), or high risk (RS ≥ 31) of disease recurrence. Low risk patients are recommended to receive endocrine-modulating therapy (tamoxifen or aromatase inhibitors) only, and high risk patients are recommended to receive both endocrine-modulating therapy and adjuvant chemotherapy [11, 13, 16]. Intermediate risk patients, while previously recommended to receive adjuvant chemotherapy, were recently shown by the large prospective TAILORx trial to receive little benefit from chemotherapy, with a notable exception for younger patients [17]. Additional studies have also validated the usefulness of ODX in patients with LN positive disease [18–20].

Several studies have suggested that ODX test results influence subsequent treatment decisions. Approximately one-third to one-half of patient-physician pairs make a change in recommended treatment following ODX, generally eschewing adjuvant chemotherapy in favor of the less toxic endocrine-modulating-only regimen [21, 22]. Despite its clinical impact, some eligible patients are not tested, with the most common reason being that ODX was not offered by the physician [23]. Physicians’ lack of familiarity with genomic testing is a known barrier to clinical implementation [24].

Qualitative and quantitative studies have examined patient and physician characteristics associated with use of ODX, yet studies examining subsequent chemotherapy use following ODX testing have primarily focused on patient characteristics [21, 22, 25–32]. In this study, we examined New Hampshire State Cancer Registry data from 2010 to 2016 to identify clinicopathological factors, patient demographics, and physician and hospital characteristics that influenced receipt of the ODX test in BC patients, the physician’s decision to recommend chemotherapy, and the receipt of adjuvant chemotherapy by the patient.

## Methods

### Data sources

The New Hampshire State Cancer Registry (NHSCR) is maintained by the State of New Hampshire Department of Health and Human Services. This is a population-based database on incident reportable cancers for all New Hampshire residents and includes patient demographics, date and mode of diagnosis, and tumor characteristics including grade and stage [33]. The NHSCR achieved the highest standard (gold) certification of data quality from the North American Association of Central Cancer Registries throughout the study period [34].

We obtained physician characteristics from two sources. The National Plan and Provider Enumeration System (NPPES) Downloadable File from the Centers for Medicare and Medicaid Services (CMS) enumerates the National Provider Identifier (NPI) for all physicians in the United States. All HIPAA-covered entities (clinicians and organizations) have been required to hold an NPI since 2007. The NPPES file is continuously updated and contains nearly 5 million records [35]. The CMS Physician Compare National Downloadable File is another resource providing general information regarding physicians caring for Medicare eligible patients in the United States [36].

### Study cohort and definitions

Our study cohort includes women residing in New Hampshire and diagnosed with breast cancer from 2010 to 2016, between the ages of 18 and 99. We excluded patients with ductal carcinoma in situ (DCIS) or unknown stage. We further excluded patients with no recorded medical oncologist in the registry. We included the characteristics of each patient’s primary medical oncologist, identified as having an NPI specialty designation in Gynecologic Oncology, Hematology, Hematology and Oncology, Medical Oncology, or Pediatric Hematology-Oncology.

### Study variables

#### Outcome variables

The NHSCR documents whether patients receive ODX and their test results. It also includes variables describing each patient’s treatment plan including whether chemotherapy was recommended, whether it was given, and whether the patient refused chemotherapy after physician recommendation. This allowed us to examine multiple outcomes: use of ODX, being recommended chemotherapy following ODX, receiving chemotherapy following ODX, and chemotherapy refusal following ODX. We further examined factors associated with receiving chemotherapy stratified by ODX RS classification (low, intermediate, high).

#### Patient variables

Patient variables include sociodemographic characteristics (patient age at diagnosis, marital status, and payer) and tumor characteristics (year of diagnosis, size, grade, LN status, hormone receptor status, and clinical stage).

#### Physician variables

Physician variables include gender, clinical experience, and patient volume. To determine years of clinical experience for each physician, the difference between the physician’s graduation year and the patient’s year of diagnosis was calculated. Patient volume was calculated as the average number of BC patients in the NHSCR data treated per year for each physician. Average patient age was calculated as the mean age at diagnosis for all patients seen by the physician in the NHSCR. A binary variable was defined to discriminate between a patient being seen by a surgical oncologist or a general surgeon.

### Statistical analysis

We first performed unadjusted analyses for all covariates. We developed multivariable logistic regression models to examine the likelihood of ODX receipt in relation to patient and provider factors. Variables found to be significant at alpha = 0.05 during unadjusted or adjusted analysis were retained for further analysis. Variables found to be non-significant in both were dropped from the final analyses. Finally, we performed hierarchical logistic regressions, specifying hospital or physician as a random effect. We identified the intraclass correlation coefficient (ICC) which quantifies the amount of clustering due to the random effect and not to the observed factors, in order to determine the contribution to the variance from the random effect, as previously reported [37–39]. Data analysis was performed with R version 3.6.0 [40].

## Results

The initial NHSCR dataset contained 10,768 unique breast cancer patients diagnosed from 2010 through 2016 (Table 1). A small number of patients (*n* = 29) received MammaPrint, a similar genomic test, and these patients were excluded from analysis. A total of 91 patients were excluded due to ineligible age or gender. Patients were then excluded if they had DCIS (*n* = 2141), unknown stage (*n* = 341), or if they did not have a recorded medical oncologist (*n* = 2536), yielding a final cohort of 5630 women (Supplemental Figure S1**)**. There were 225 unique medical oncologists treating the patients in the cohort (Table 2).

**Table 1:** Statistics of BC patients in New Hampshire 2010–2016

Variable	ODX Not Given (***n*** = 4118)	ODX Given (***n*** = 1512)	Total (***n*** = 5630)	***P***-Value
**Patient Age at Diagnosis (Years)**				< 0.01**
< 50	7870 (18.9%)	317 (21.0%)	1097 (19.5%)	
50–59	974 (23.7%)	447 (29.6%)	1421 (25.2%)	
60–69	1166 (28.3%)	518 (34.3%)	1684 (29.9%)	
> 69	1198 (29.1%)	230 (15.2%)	1428 (25.4%)	
**Marital Status**				< 0.01**
Single	1620 (39.3%)	464 (30.7%)	2084 (37.0%)	
Married	2391 (58.1%)	1010 (66.8%)	3401 (60.4%)	
Unknown	107 (2.6%)	38 (2.5%)	145 (2.6%)	
**Payer**				< 0.01**
Self-Pay	86 (2.1%)	18 (1.2%)	104 (1.8%)	
Public	1901 (46.2%)	516 (34.1%)	2417 (42.9%)	
Private	1659 (40.3%)	790 (52.2%)	2449 (43.5%)	
Unknown	472 (11.5%)	188 (12.4%)	660 (11.7%)	
**Year of Diagnosis**				0.14
2010	526 (12.8%)	172 (11.4%)	698 (12.4%)	
2011	577 (14.0%)	198 (13.1%)	775 (13.8%)	
2012	528 (12.8%)	185 (12.2%)	713 (12.7%)	
2013	580 (14.1%)	189 (12.5%)	769 (13.7%)	
2014	613 (14.9%)	242 (16.0%)	855 (15.2%)	
2015	668 (16.2%)	269 (17.8%)	937 (16.6%)	
2016	626 (15.2%)	257 (17.0%)	883 (15.7%)	
**Tumor Size (mm)**				< 0.01**
0.1–19	2390 (58.0%)	1023 (67.7%)	3413 (60.6%)	
20–39	981 (23.8%)	417 (27.6%)	1398 (24.8%)	
> 40	627 (15.2%)	64 (4.2%)	691 (12.3%)	
Unknown	120 (2.9%)	8 (0.5%)	128 (2.3%)	
**Tumor Grade**				< 0.01**
I	854 (20.7%)	398 (26.3%)	1252 (22.2%)	
II	1719 (41.7%)	844 (55.8%)	2563 (45.5%)	
III / IV	1403 (34.1%)	265 (17.5%)	1668 (29.6%)	
Unknown	142 (3.4%)	5 (0.3%)	147 (2.6%)	
**LN Status**				< 0.01**
Negative	2289 (55.6%)	1196 (79.1%)	3485 (61.9%)	
Positive	1257 (30.5%)	284 (18.8%)	1541 (27.4%)	
Unknown	572 (13.9%)	32 (2.1%)	604 (10.7%)	
**ER/PR/HER2 Status**				< 0.01**
HR+/HER2-	2663 (64.7%)	1446 (95.6%)	4109 (73.0%)	
Other	1270 (30.8%)	41 (2.7%)	1311 (23.3%)	
Unknown	185 (4.5%)	25 (2.5%)	210 (3.7%)	
**Clinical Stage**				< 0.01**
1	2152 (52.3%)	1034 (68.4%)	3186 (56.6%)	
2	1166 (28.3%)	453 (30.0%)	1619 (28.8%)	
3 / 4	800 (19.4%)	25 (1.7%)	825 (14.7%)	
**ODX Eligible**^a^	1472 (35.7%)	1132 (74.9%)	2604 (46.3%)	< 0.01**
**MD Gender**				0.34
Female	2212 (53.7%)	790 (52.2%)	3002 (53.3%)	
Male	1906 (46.3%)	722 (47.8%)	2628 (46.7%)	
**MD Clinical Experience (Years)**				0.08
< 10	169 (4.1%)	58 (3.8%)	227 (4.0%)	
10–19	1774 (43.1%)	604 (39.9%)	2378 (42.2%)	
20–29	1254 (30.5%)	511 (33.8%)	1765 (31.3%)	
> 29	921 (22.4%)	339 (22.4%)	1260 (22.4%)	
**Surgical Specialty**				0.72
General Surgeon	3582 (87.0%)	1309 (86.6%)	4891 (86.9%)	
Surgical Oncologist	536 (13.0%)	203 (13.4%)	739 (13.1%)	

*P*-values were calculated using chi-square test for categorical variables

* significant at the 0.05 level

** significant at the 0.01 level

^a^ODX eligible patients are defined as stage 1 or 2, LN negative, and HR+/HER2-

**Table 2:** Physician summary statistics

	Total (***n*** = 225)
**Gender**	
Female	121 (53.8%)
Male	104 (46.2%)
**Clinical Experience (Years)** (at time of treating first BC patient in cohort)	
< 10	42 (18.7%)
10–19	68 (30.2%)
20–29	72 (32.0%)
> 29	43 (19.1%)
**Graduation Year**	
1960s	6 (2.7%)
1970s	23 (10.2%)
1980s	72 (32.0%)
1990s	64 (28.4%)
2000s	58 (25.7%)
2010s	2 (0.9%)
**Patient Volume**^a^	
Mean (Standard Deviation)	5.14 (8.94)
**Average Patient Age**	
< 65 Years	165 (73.3%)
> 65 Years	60 (26.7%)

^a^BC patients seen per year

### Receiving ODX

Of the total cohort, 1512 (26.9%) patients were tested with ODX. Over the course of the study period, overall use of ODX increased from 24.6% in 2010 to 29.1% in 2016 (*p* = 0.05) (Table 1). In unadjusted analyses, we found patient age, marital status, payer, tumor grade, LN status, tumor size, clinical stage, and being seen by an oncologist with an older average patient age were significantly associated with receiving ODX (Table S1). In the adjusted analysis, patient age, marital status, tumor grade, LN status, tumor size, and clinical stage contributed significantly to the model (Table 3). We then examined patient and physician characteristics associated with ODX testing specifically among patients eligible for ODX. Of the 2604 patients eligible for ODX, defined as stage 1 or 2, LN negative, and HR+/HER2-, 1132 (43.5%) received the test. ODX use in eligible patients ranged from 42.5% in 2010 to 45.4% in 2016 (*p* = 0.50). In the unadjusted analysis, patient age, marital status, tumor grade, tumor size, tumor stage, physician gender, physician patient volume, and being seen by an oncologist with an older average patient age were significantly associated with ODX use (Table S1). Only patient age, marital status, tumor grade, and tumor size contributed significantly to the adjusted model (Table S2).

**Table 3:** Multivariable regression odds ratios for receiving ODX

Variable	Odds Ratio (95% CI)	***P***-Value
**Year of Diagnosis**		
2010	Ref	Ref
2011	1.00 (0.76–1.31)	0.99
2012	1.00 (0.76–1.31)	0.99
2013	1.00 (0.76–1.31)	0.98
2014	1.05 (0.80–1.36)	0.73
2015	1.05 (0.80–1.36)	0.73
2016	1.09 (0.83–1.43)	0.54
**Patient Age at Diagnosis (Years)**		
< 50	Ref	Ref
50–59	1.04 (0.86–1.27)	0.66
60–69	0.97 (0.79–1.18)	0.75
> 69	0.45 (0.35–0.58)	< 0.01**
**Marital Status**		
Single, Divorced, Widowed	Ref	Ref
Married	1.22 (1.05–1.41)	< 0.01**
Unknown	1.12 (0.73–1.73)	0.59
**Grade**		
I	Ref	Ref
II	1.22 (1.03–1.43)	0.02*
III/IV	0.43 (0.35–0.53)	< 0.01**
Unknown	0.16 (0.06–0.42)	< 0.01**
**LN Status**		
Negative	Ref	Ref
Positive	0.70 (0.58–0.86)	< 0.01**
Unknown	0.21 (0.14–0.31)	< 0.01**
**Tumor Size (mm)**		
0.1–19	Ref	Ref
20–39	1.69 (1.35–2.12)	< 0.01**
> 40	0.88 (0.61–1.27)	0.48
Unknown	0.92 (0.39–2.13)	0.84
**Clinical Stage**		
1	Ref	Ref
2	0.80 (0.63–1.03)	0.08
3 / 4	0.10 (0.06–0.17)	< 0.01**
**MD Clinical Experience (Years)**		
< 10	Ref	Ref
10–19	0.89 (0.61–1.29)	0.53
20–29	1.05 (0.71–1.57)	0.79
> 29	1.07 (0.70–1.61)	0.76
**MD Gender**		
Female	Ref	Ref
Male	0.98 (0.79–1.22)	0.88
**Patient Volume**	1.00 (0.99–1.01)	0.48
**Average Patient Age**		
< 65 Years	Ref	Ref
> 65 Years	0.71 (0.49–1.03)	0.06
**Surgical Specialty**		
General Surgeon	Ref	Ref
Surgical Oncologist	0.86 (0.70–1.06)	0.15

* significant at the 0.05 level

** significant at the 0.01 level

### Chemotherapy recommendation

Chemotherapy was recommended for 2701 (48.0%) patients in the breast cancer cohort and 459 (30.4%) of patients who received ODX. In the unadjusted analyses, we found year of diagnosis, patient age, tumor grade, LN status, tumor size, clinical stage, physician gender, clinical experience, physician patient volume, and ODX RS stratification to be significantly associated with a recommendation for chemotherapy (Table S1). In the adjusted model, year of diagnosis, patient age, tumor grade, LN status, tumor size, physician clinical experience, physician gender, physician patient volume, and ODX RS stratification were significantly associated with a recommendation for chemotherapy. Notably, we found that patients were less likely to be recommended chemotherapy if they were seen by a male (compared to female) medical oncologist (OR = 0.50 (95% CI = 0.34–0.74), *p* < 0.01). Compared with patients treated by medical oncologists with fewer than 10 years of clinical experience, patients treated by medical oncologists with more clinical experience were more likely to be recommended chemotherapy (20–29 years: OR = 4.05 (95% CI = 1.57–10.43), *p* < 0.01; > 29 years: OR = 4.48 (95% CI = 1.68–11.95), *p* < 0.01) (Table 4).

**Table 4:** Multivariable regression odds ratios for chemotherapy recommendation following ODX testing

Variable	Odds Ratio (95% CI)	***P***-Value
**Year of Diagnosis**		
2010	Ref	Ref
2011	0.87 (0.48–1.60)	0.66
2012	0.65 (0.34–1.24)	0.18
2013	0.73 (0.39–1.36)	0.31
2014	0.66 (0.36–1.19)	0.16
2015	0.44 (0.24–0.82)	< 0.01**
2016	0.51 (0.27–0.96)	0.04*
**Patient Age at Diagnosis (Years)**		
< 50	Ref	Ref
50–59	0.73 (0.48–1.10)	0.13
60–69	0.39 (0.25–0.61)	< 0.01**
> 69	0.35 (0.18–0.66)	< 0.01**
**Grade**		
I	Ref	Ref
II	1.73 (1.16–2.59)	< 0.01**
III/IV	3.55 (2.17–5.83)	< 0.01**
Unknown	2.61 (0.34–20.30)	0.35
**LN Status**		
Negative	Ref	Ref
Positive	3.54 (2.29–5.46)	< 0.01**
Unknown	1.26 (0.39–4.08)	0.69
**Tumor Size (mm)**		
0.1–19	Ref	Ref
20–39	1.51 (0.94–2.45)	0.08
> 40	4.36 (1.98–9.62)	< 0.01**
Unknown	1.03 (0.18–6.05)	0.98
**Clinical Stage**		
1	Ref	Ref
2	1.50 (0.90–2.50)	0.11
3 / 4	2.72 (0.86–8.65)	0.08
**MD Clinical Experience (Years)**		
< 10	Ref	Ref
10–19	1.89 (0.74–4.81)	0.17
20–29	4.05 (1.57–10.43)	< 0.01**
> 29	4.48 (1.68–11.95)	< 0.01**
**MD Gender**		
Female	Ref	Ref
Male	0.50 (0.34–0.74)	< 0.01**
**Patient Volume**	1.01 (1.01–1.03)	0.04*
**Average Patient Age**		
< 65 Years	Ref	Ref
> 65 Years	0.72 (0.34–1.56)	0.40
**ODX RS Classification**		
Low	Ref	Ref
Intermediate	12.30 (8.70–17.38)	< 0.01**
High	233.08 (95.40–569.42)	< 0.01**

* significant at the 0.05 level

** significant at the 0.01 level

### Receiving chemotherapy

**Fig. 1:**
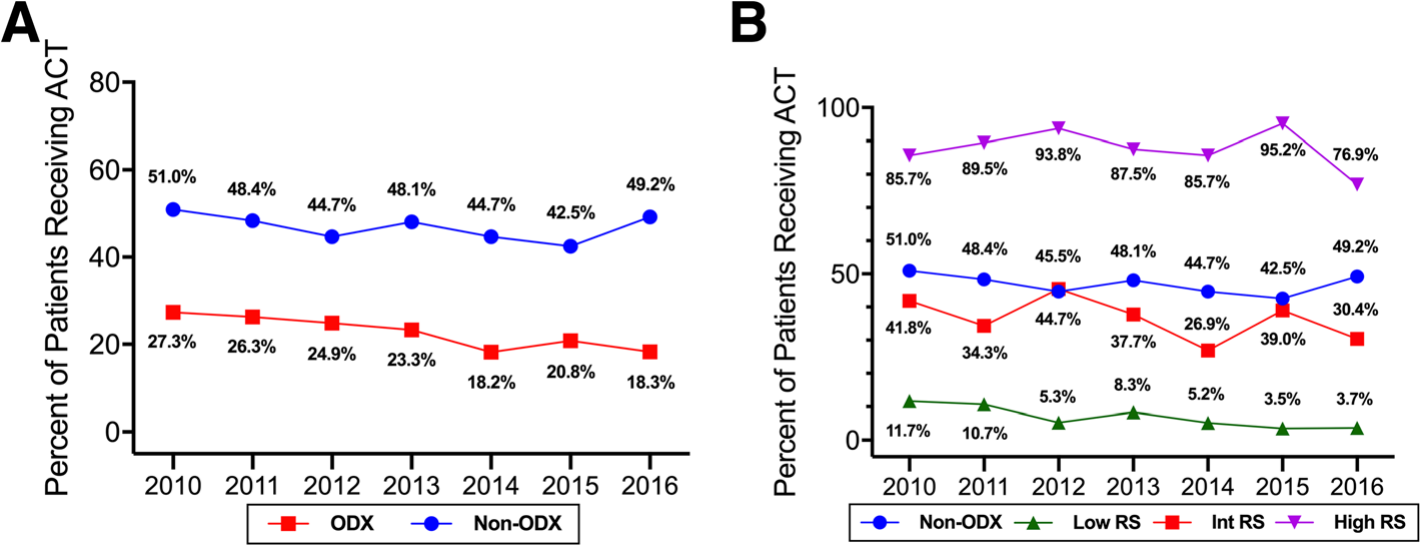
**a** Trends in chemotherapy receipt of patients receiving and not receiving ODX (**b**) Trends in chemotherapy receipt by RS stratification in ODX patients. ACT = adjuvant chemotherapy

Receipt of chemotherapy was documented in 2264 (40.2%) patients in the breast cancer cohort, and 336 (22.2%) of patients who received ODX. Receipt of chemotherapy among patients who did not receive ODX remained relatively unchanged during the study period (− 3.53% relative change from 2010 to 2016 (*p* = 0.37)). However, in patients who received ODX, chemotherapy use decreased from 27.3% in 2010 to 18.3% in 2016, a relative change of − 33.0% (*p* = 0.02) (Fig. 1a**)**. In unadjusted analyses, the significant factors associated with chemotherapy receipt following ODX testing were year of diagnosis, patient age, payer, tumor grade, LN status, tumor size, clinical stage, physician’s average patient age, and ODX RS stratification (Table S1). In the multivariable model, year of diagnosis, patient age, tumor grade, LN status, tumor size, clinical stage, physician clinical experience, physician gender, and ODX RS stratification were significantly associated with patient receipt of chemotherapy (Table S3).

### Receiving chemotherapy by ODX risk classification

We then stratified the ODX patients by their RS (low, intermediate, high) and developed a multivariable model for each stratum. Low RS patients comprised 60.6% of the ODX population (*n* = 917) and 6.4% of these patients received chemotherapy. Chemotherapy use decreased from 11.7% in 2010 to 3.7% in 2016 for a relative change of − 68.4% (*p* = 0.02) (Fig. 1b). Low risk patients were less likely to receive chemotherapy if they were older (60–69 years vs. < 50 years: OR = 0.17 (95% CI = 0.06–0.49), *p* < 0.01; > 69 years vs. < 50 years: OR = 0.08 (95% CI = 0.02–0.45), *p* < 0.01) and were more likely to receive chemotherapy for higher grade (grade III/IV vs. grade I: OR = 7.80 (95% CI = 2.62–23.27), *p* < 0.01), positive compared to negative LN status (OR = 5.84 (95% CI = 2.61–13.05), *p* < 0.01), higher clinical stage (Stage 2 vs. Stage 1: OR = 2.96 (95% CI = 1.10–7.98) *p* = 0.03; Stage 3/4 vs. Stage 1: OR = 6.22 (95% CI = 1.18–32.74), p = 0.03) and larger tumors (> 40 mm vs. 0.1-19 mm: OR = 8.16 (95% CI = 2.37–28.06), *p* < 0.01). In addition, chemotherapy receipt was less likely among patients treated by male (vs. female) medical oncologists (OR = 0.39 (95% = 0.17–0.88), *p* = 0.02) (Table 5).

**Table 5:** Multivariable regression odds ratios for receiving chemotherapy stratified by low and intermediate ODX RS

Variable	Odds Ratio (95% CI) Low RS	***P***-Value	Odds Ratio (95% CI) Intermediate RS	***P***-Value
**Year of Diagnosis**				
2010	Ref	Ref	Ref	Ref
2011	0.68 (0.21–2.17)	0.50	1.17 (0.45–3.04)	0.75
2012	0.48 (0.14–1.72)	0.25	2.23 (0.82–6.06)	0.11
2013	0.34 (0.10–1.17)	0.08	1.10 (0.41–2.95)	0.84
2014	0.40 (0.12–1.39)	0.14	0.67 (0.26–1.72)	0.40
2015	0.21 (0.05–0.86)	0.03*	1.07 (0.41–2.76)	0.89
2016	0.25 (0.06–0.94)	0.04*	1.10 (0.39–3.10)	0.85
**Patient Age at Diagnosis (Years)**				
< 50	Ref	Ref	Ref	Ref
50–59	0.67 (0.30–1.50)	0.32	0.59 (0.31–1.13)	0.11
60–69	0.17 (0.06–0.49)	< 0.01**	0.29 (0.14–0.59)	< 0.01**
> 69	0.08 (0.02–0.45)	< 0.01**	0.10 (0.03–0.33)	< 0.01**
**Grade**				
I	Ref	Ref	Ref	Ref
II	1.48 (0.58–3.73)	0.40	2.00 (1.06–3.80)	0.03*
III/IV	7.80 (2.62–23.27)	< 0.01**	2.37 (1.10–5.11)	0.02*
Unknown	0.00 (0.00-Inf)	0.99	0.00 (0.00-Inf)	0.99
**LN Status**				
Negative	Ref	Ref	Ref	Ref
Positive	5.84 (2.61–13.05)	< 0.01**	1.93 (0.92–4.05)	0.08
Unknown	2.91 (0.25–34.27)	0.38	0.50 (0.04–6.96)	0.60
**Tumor Size (mm)**				
0.1–19	Ref	Ref	Ref	Ref
20–39	0.96 (0.38–2.41)	0.93	1.26 (0.55–2.88)	0.58
> 40	8.16 (2.37–28.06)	< 0.01**	0.94 (0.19–4.62)	0.94
Unknown	0.00 (0.00-Inf)	0.99	0.79 (0.07–9.12)	0.85
**Clinical Stage**				
1	Ref	Ref	Ref	Ref
2	2.96 (1.10–7.98)	0.03*	2.59 (1.04–6.47)	0.04*
3 / 4	6.22 (1.18–32.74)	0.03*	0.36 (0.01–9.58)	0.53
**MD Clinical Experience (Years)**				
< 10	Ref	Ref	Ref	Ref
10–19	2.56 (0.26–25.28)	0.41	0.75 (0.21–2.64)	0.65
20–29	4.31 (0.44–41.94)	0.20	1.61 (0.44–5.88)	0.46
> 29	7.71 (0.75–79.43)	0.08	2.02 (0.55–7.48)	0.28
**MD Gender**				
Female	Ref	Ref	Ref	Ref
Male	0.39 (0.17–0.88)	0.02*	0.73 (0.42–1.27)	0.26
**Patient Volume**	0.98 (0.95–1.00)	0.08	1.01 (0.99–1.02)	0.40
**Average Patient Age**				
< 65 Years	Ref	Ref	Ref	Ref
> 65 Years	0.59 (0.13–2.74)	0.50	0.76 (0.23–2.53)	0.65
**ODX RS**	0.98 (0.96–1.00)	0.15	1.33 (1.22–1.44)	< 0.01**

* significant at the 0.05 level

** significant at the 0.01 level

Intermediate RS patients comprised 31.0% of the ODX population (*n* = 469), and 35.6% of the intermediate RS patients received chemotherapy. Chemotherapy use in this group decreased from 41.8 to 30.4%, a relative change of − 27.3% (*p* = 0.37) (Fig. 1b). In multivariable models, chemotherapy was less likely in older patients compared to those less than 50 years (60–69 years: OR = 0.29 (95% CI = 0.14–0.59), *p* < 0.01; > 69 years: OR 0.10 (95% CI = 0.03–0.33), *p* < 0.01). Chemotherapy was more likely in those with higher tumor grade compared to grade I (Grade II: OR = 2.00 (95% CI = 1.06–3.80), *p* = 0.03; Grade III/IV: OR = 2.37 (95% CI = 1.10–5.11), *p* = 0.02), and in those with higher clinical stage (Stage 2 vs. Stage 1: OR = 2.59 (95% CI = 1.04–6.47), *p* = 0.04) and higher ODX RS (OR = 1.33 (95% CI = 1.22–1.44), *p* < 0.01) (Table 5).

High RS patients comprised 8.3% of the ODX population (*n* = 126) and 87.3% of these patients received chemotherapy. Chemotherapy use in the high RS group decreased from 85.7 to 76.9% between 2010 and 2016 (*p* = 0.88) (Fig. 1b). Of all the high RS patients, 61.9% had grade 3/4 tumors, 81.0% were LN negative, and 63.5% had Stage 1 BC. The high RS classification model failed to converge.

### Chemotherapy refusal

A total of 375 patients were reported to have refused a recommended course of adjuvant chemotherapy, 109 of these having received ODX. Of those tested with ODX who later refused recommended chemotherapy, the majority were in intermediate RS range (56.0%), were stage 1 (57.8%), LN negative (66.1%), and had tumors that were grade II (60.6%). In the multivariable model, older patients were more likely to refuse chemotherapy compared to patients less than 50 years (> 69 years: OR = 5.62 (95% CI = 1.72–18.39), *p* < 0.01). Patients were less likely to refuse recommended adjuvant chemotherapy following ODX testing if they had intermediate or high ODX RS stratification, when compared with low RS (Intermediate: OR 0.30 (95% CI = 0.15–0.60), *p* < 0.01; High: OR 0.04 (95% CI = 0.01–0.13), *p* < 0.01). In addition, patients being seen by higher volume oncologists were more likely to refuse chemotherapy (OR 1.02 (95% CI = 1.01–1.04), *p* = 0.04) (Table S4).

### Between-physician and between-hospital variation

Hierarchical modeling for each outcome using hospital and physician as the random effect allowed us to determine the proportion of total variance in clinical decisions that is due to variation between physicians and hospitals. For each model, we calculated the ICC in order to measure the correlation of clinical decisions within physicians or hospitals (Table 6). Overall, between-physician variation accounted for a greater proportion of variance than between-hospital variation. Clustering within treating physicians and hospitals was most pronounced for patients receiving a low ODX RS score: clustering within physicians and within hospitals accounted for 33 and 14% of the total variance in chemotherapy use, respectively. For all patients tested with ODX, clustering within physicians and within hospitals accounted for 18 and 4% of variation in receiving chemotherapy, respectively.

**Table 6:** ICC for receiving ODX and being recommended, receiving, and refusing chemotherapy

Clinical Decision	Hospital-level ICC	Physician-level ICC
Receiving ODX for all patients	0.02	0.07
Receiving ODX for eligible patients^a^	0.04	0.11
Recommending chemotherapy after ODX	0.06	0.17
Receiving chemotherapy after ODX	0.04	0.18
Receiving chemotherapy after Low ODX RS	0.14	0.33
Receiving chemotherapy after Int ODX RS	0.03	0.18
Refusing chemotherapy after ODX	0.09	0.27

^a^ODX eligible patients are defined as stage 1 or 2, LN negative, and HR+/HER2-

## Discussion

Increasing use of ODX is expected to spare low risk patients the short- and long-term adverse effects of adjuvant chemotherapy, while still treating the patients who are most likely to benefit [41]. Previous studies using the National Cancer Data Base report utilization of ODX of 45.7 to 54.0% among eligible patients, which is similar to our finding of 43.5%; however, these rates suggest a national underutilization of ODX [27, 42]. Between 2010 and 2016, ODX use increased among patients with BC in New Hampshire, and low and intermediate risk patients were more often spared chemotherapy while higher risk patients continued to receive chemotherapy at higher rates. These findings suggest that physicians were following ODX recommendations as they became available and sparing chemotherapy in patients who were unlikely to receive any benefit.

Previously identified factors associated with utilization of ODX fall under patient, physician, and organizational level factors, among which our study attempted to differentiate [43]. Our final models indicate that patients with earlier stage, LN negative BC were more likely to be prescribed the test. Patient-level factors for which we did not account but which literature suggests play a role in shared decision-making include education, decision-making style, and attitude towards genetic testing and chemotherapy [23, 44]. Cost is unlikely to have been a major barrier during our study period, as ODX testing has been covered by CMS and most private payers for eligible patients since 2006–2008 [27, 45]. In our study, we did not find physician gender or clinical experience to be associated with use of ODX. Previous work identified physician awareness and familiarity with genomic testing as a barrier to uptake [24]. This is reflected by oncologists reporting a desire to receive additional education regarding genomic tests [46]. Physicians also cite ODX marketing, medical/insurance guidelines, and use among peers as factors contributing to utilization of ODX in their practice [43].

We found that patients who received ODX were more likely to be recommended for chemotherapy if they were younger and had later stage, LN positive BC, and higher ODX RS, consistent with previous work [47]. We observed that the association between absolute RS and odds of chemotherapy treatment to be strongest among intermediate risk patients. Other interesting patterns reflecting the influence of physician characteristics on chemotherapy use following ODX stand out. Patients tested with ODX were significantly more likely to be recommended chemotherapy when treated by physicians with 20 or more years of clinical experience. This may represent aspects of the doctor-patient relationship as well as acceptance of RS score guidelines and engrained practice patterns, as these physicians would have been in practice when guidelines recommending chemotherapy for all patients were established [3, 4]. We observed that female physicians were more likely to recommend and prescribe chemotherapy for all ODX patients, including low risk patients. Additional work to understand the differences in preferences of oncologists accounting for gender and clinical experience may be warranted to reduce variation in treatment decisions following ODX test results, especially given the potential concern of overtreatment among low risk patients.

Our hierarchical models demonstrate the significant heterogeneity in chemotherapy treatment decisions following ODX testing among hospitals and physicians. In this respect, variation between hospitals seemed to be less pronounced than variation between physicians. These results raise questions regarding the extent to which unmeasured physician characteristics impact their interpretation of ODX results and subsequent treatment decisions. The variation identified by the physician ICCs may represent differences in physician training, personal experience and familiarity with genetic tests, and the perceived value of the test, all of which have been reported previously [24, 48].

Our study has several limitations. New Hampshire has a predominantly white and rural population, so our findings may not be generalizable to other states or regions with different patient and provider sociodemographic characteristics. We did not have access to detailed medical records and thus could not analyze outcomes, such as disease-free survival following ODX testing or by treatment modality. Patients missing data on medical oncologists was a limitation, which could also be better addressed in a study that links the registry data to electronic health records. Coder reliability and misclassification are a known issue when analyzing registry data as evidenced by the recent reliability study conducted by the Surveillance, Epidemiology, and End Results (SEER) Program [49]. We lacked data on whether the physician specializes in breast cancer care, which could influence their use of ODX. Finally, due to the observational nature of our study, the associations we identified cannot be interpreted as causal.

## Conclusions

In conclusion, these findings indicate potential opportunities to implement interventions and target physicians regarding ODX and adjuvant chemotherapy use in order to reduce variation in patient care. This is especially important, and challenging, as ODX recommendations continue to evolve in light of new findings, such as those from the TAILORx trial [17]. Moreover, the utility of ODX has now extended to a stage modifier according to the American Joint Committee on Cancer staging manual [50]. Future work evaluating guideline-concordant changes in ODX testing and adjuvant chemotherapy prescribing patterns following new guidelines will shed light on physician awareness and adaptability regarding implementation of genomic tests in cancer care. Additional physician training in the availability of genomic tests, interpreting genetic tests, and methods to convey the benefits and results of the tests may be beneficial to increase utilization [23, 48].

## Supplementary information

**Additional file 1.**

## Data Availability

The data that support the findings of this study are available from the New Hampshire Department of Health and Human Resources, but restrictions apply to the availability of the data, which were used with permission for the current study, and so are not publicly available. Data are, however, available from the authors upon reasonable request and with permission of the New Hampshire Department of Health and Human Resources.

## Abbreviations

ODX: Oncotype DX
BC: Breast cancer
LN: Lymph node
HR: Hormone receptor
RS: Recurrence score
OR: Odds ratio
CI: Confidence interval
ICC: Intraclass clustering coefficient
NHSCR: New Hampshire State Cancer Registry
CMS: Center for Medicare and Medicaid Services
NPI: National Provider Identifier
