# Supplementary material for: Patient and physician factors associated with Oncotype DX and adjuvant chemotherapy utilization for breast cancer patients in New Hampshire, 2010–2016
Source: BMC Cancer. 2020 Sep 3;20:847. doi: 10.1186/s12885-020-07355-6 (PMC7650301; doi:10.1186/s12885-020-07355-6)
Supplement: Supplementary file 1 — Additional file 1. [file 12885_2020_7355_MOESM1_ESM.pdf]

**SUPPLEMENTAL MATERIAL**

# Patient and physician factors associated with Oncotype DX and adjuvant chemotherapy utilization for breast cancer patients in New Hampshire, 2010-2016

Thomas M. Schwedhelm[1], Judy R. Rees[2,3], Tracy Onega[1,3,4], Ronnie J. Zipkin[1], Andrew Schaefer[4], Mario O Celaya[2,3], Erika L. Moen[1,4*]

[1]Department of Biomedical Data Science, Dartmouth Geisel School of Medicine, Lebanon, NH
[2]New Hampshire State Cancer Registry, Lebanon, NH
[3]Department of Epidemiology, Dartmouth Geisel School of Medicine, Lebanon, NH
[4]The Dartmouth Institute for Health Policy and Clinical Practice, Lebanon, NH

Short title: Oncotype DX and chemotherapy trends in New Hampshire

*Corresponding author
Erika L. Moen
1 Medical Center Dr
Lebanon, NH 03756
Ph: (603) 650-1976
E-mail: Erika.L.Moen@dartmouth.edu
Fax: (603) 650-1900

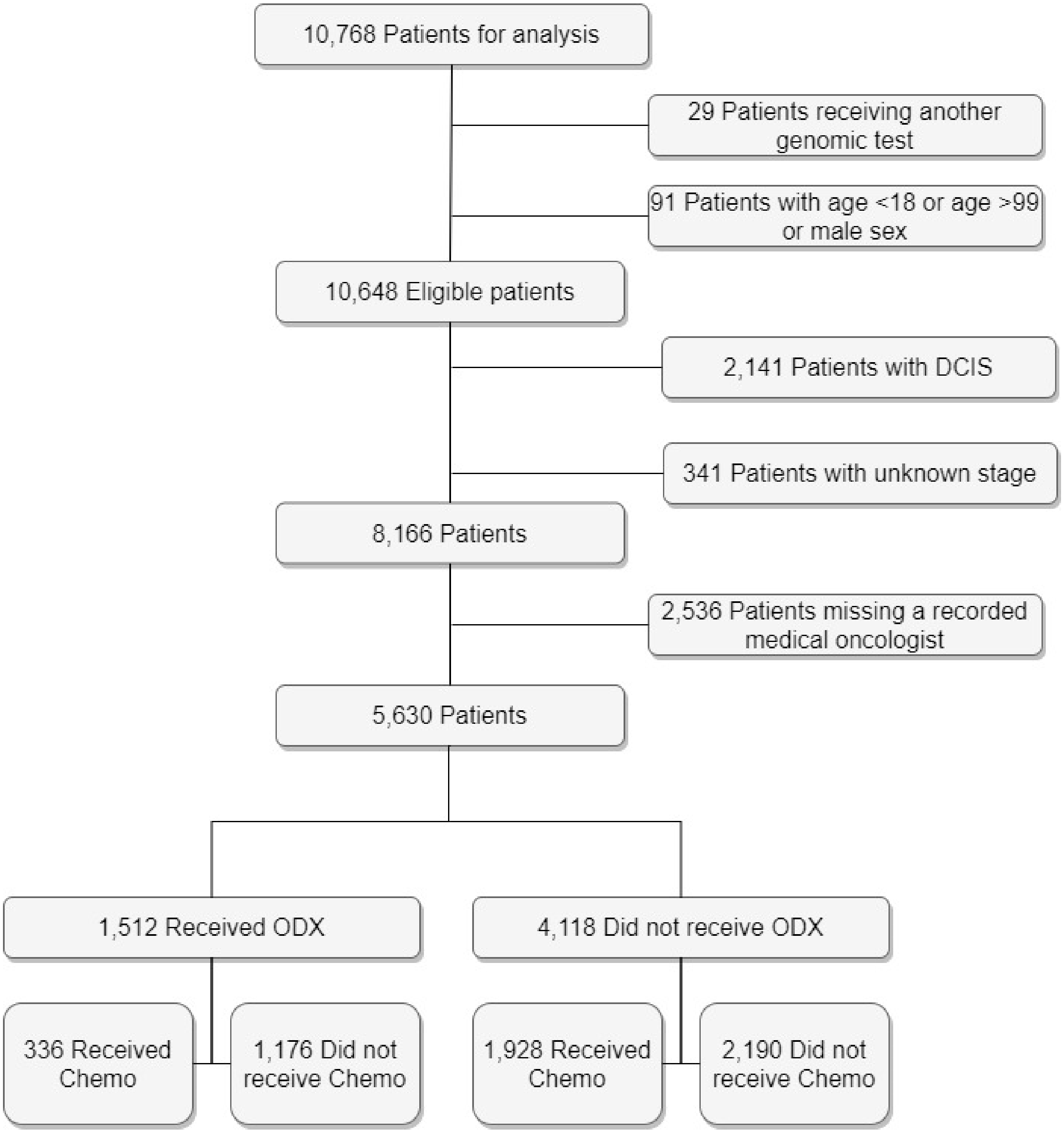

**Fig S1** Dataset flow diagram delineating exclusion criteria

**Table S1** Unadjusted analyses for receiving ODX, receiving ODX among ODX eligible patients†, being recommended chemotherapy after ODX, and receiving chemotherapy following ODX.

| Variable | All Patients ODX Odds Ratio (95% CI) | P-Value | ODX Eligible Patients† ODX Odds Ratio (95% CI) | P-Value | Chemotherapy Recommendation Odds Ratio (95% CI) | P-Value | Chemotherapy Receipt Odds Ratio (95% CI) | P-Value |
|---|---|---|---|---|---|---|---|---|
| **Year of Diagnosis** | | | | | | | | |
| 2010 | Ref | Ref | Ref | Ref | Ref | Ref | Ref | Ref |
| 2011 | 1.05 (0.82-1.34) | 0.69 | 1.09 (0.79-1.51) | 0.58 | 0.96 (0.63-1.49) | 0.87 | 0.95 (0.59-1.52) | 0.82 |
| 2012 | 1.07 (0.84-1.37) | 0.57 | 0.91 (0.66-1.26) | 0.56 | 0.73 (0.47-1.15) | 0.17 | 0.88 (0.54-1.43) | 0.60 |
| 2013 | 1.00 (0.78-1.27) | 0.98 | 0.94 (0.68-1.29) | 0.68 | 0.75 (0.48-1.17) | 0.19 | 0.81 (0.50-1.31) | 0.38 |
| 2014 | 1.21 (0.96-1.52) | 0.10 | 1.14 (0.84-1.55) | 0.40 | 0.71 (0.47-1.09) | 0.11 | 0.59 (0.37-0.95) | 0.03* |
| 2015 | 1.23 (0.98-1.55) | 0.07 | 1.05 (0.78-1.42) | 0.72 | 0.55 (0.36-0.84) | <0.01** | 0.70 (0.44-1.10) | 0.12 |
| 2016 | 1.26 (1.00-1.58) | 0.05 | 1.12 (0.83-1.53) | 0.45 | 0.64 (0.42-0.98) | 0.04* | 0.60 (0.37-0.95) | 0.03* |
| **Patient Age at Diagnosis (Years)** | | | | | | | | |
| <50 | Ref | Ref | Ref | Ref | Ref | Ref | Ref | Ref |
| 50-59 | 1.13 (0.95-1.35) | 0.17 | 0.84 (0.65-1.08) | 0.17 | 0.86 (0.63-1.16) | 0.31 | 0.88 (0.64-1.22) | 0.45 |
| 60-69 | 1.09 (0.92-1.30) | 0.29 | 0.60 (0.47-0.77) | <0.01** | 0.59 (0.43-0.80) | <0.01** | 0.56 (0.40-0.78) | <0.01** |
| >69 | 0.47 (0.39-0.57) | <0.01** | 0.21 (0.16-0.27) | <0.01** | 0.50 (0.34-0.74) | <0.01** | 0.29 (0.18-0.48) | <0.01** |
| **Marital Status** | | | | | | | | |
| Single, Divorced, Widowed | Ref | Ref | Ref | Ref | Ref | Ref | Ref | Ref |
| Married | 1.47 (1.30-1.68) | <0.01** | 1.65 (1.39-1.96) | <0.01** | 0.82 (0.64-1.04) | 0.10 | 1.00 (0.76-1.31) | 0.99 |
| Unknown | 1.24 (0.84-1.84) | 0.27 | 1.09 (0.65-1.82) | 0.75 | 1.48 (0.74-2.94) | 0.25 | 1.64 (0.79-3.41) | 0.18 |
| **Payer** | | | | | | | | |
| Self-Pay | Ref | Ref | Ref | Ref | Ref | Ref | Ref | Ref |
| Unknown | 1.90 (1.10-3.29) | 0.02* | 1.19 (0.51-2.75) | 0.68 | 0.96 (0.34-2.74) | 0.94 | 0.72 (0.25-2.08) | 0.54 |
| Public | 1.30 (0.77-2.20) | 0.32 | 0.58 (0.26-1.32) | 0.19 | 0.68 (0.25-1.89) | 0.45 | 0.36 (0.13-0.99) | 0.04* |
| Private | 2.28 (1.35-3.85) | <0.01** | 1.49 (0.66-3.37) | 0.32 | 0.99 (0.36-2.71) | 0.98 | 0.69 (0.25-1.89) | 0.46 |
| **Grade** | | | | | | | | |
| I | Ref | Ref | Ref | Ref | Ref | Ref | Ref | Ref |
| II | 1.05 (0.91-1.22) | 0.48 | 1.83 (1.53-2.19) | <0.01** | 1.90 (1.39-2.58) | <0.01** | 1.90 (1.32-2.75) | <0.01** |
| III/IV | 0.41 (0.34-0.49) | <0.01** | 1.92 (1.49-2.46) | <0.01** | 7.07 (4.90-10.20) | <0.01** | 8.57 (5.71-12.86) | <0.01** |
| Unknown | 0.08 (0.03-0.19) | <0.01** | 0.35 (0.12-1.05) | 0.06 | 3.29 (0.52-20.85) | 0.20 | 0.00 (0.00-Inf) | 0.97 |
| **LN Status** | | | | | | | | |
| Negative | Ref | Ref | | | Ref | Ref | Ref | Ref |
| Positive | 0.43 (0.37-0.50) | <0.01** | | | 2.10 (1.60-2.76) | <0.01** | 1.79 (1.33-2.40) | <0.01** |
| Unknown | 0.11 (0.07-0.16) | <0.01** | | | 1.86 (0.89-3.86) | 0.09 | 1.33 (0.58-3.04) | 0.49 |
| **Tumor Size (mm)** | | | | | | | | |
| 0.1-19 | Ref | Ref | Ref | Ref | Ref | Ref | Ref | Ref |
| 20-39 | 0.99 (0.86-1.14) | 0.92 | 2.98 (2.41-3.70) | <0.01** | 1.74 (1.36-2.23) | <0.01** | 1.71 (1.30-2.24) | <0.01** |
| >40 | 0.24 (0.18-0.31) | <0.01** | 1.04 (0.66-1.62) | 0.88 | 3.04 (1.81-5.12) | <0.01** | 2.60 (1.51-4.46) | <0.01** |
| Unknown | 0.16 (0.07-0.32) | <0.01** | 0.63 (0.12-3.39) | 0.59 | 1.72 (0.40-7.45) | 0.46 | 1.44 (0.28-7.44) | 0.66 |
| **Clinical Stage** | | | | | | | | |
| 1 | Ref | Ref | Ref | Ref | Ref | Ref | Ref | Ref |
| 2 | 0.81 (0.71-0.92) | <0.01** | 2.03 (1.64-2.50) | <0.01** | 2.07 (1.63-2.62) | <0.01** | 2.02 (1.56-2.62) | <0.01** |
| 3 / 4 | 0.07 (0.04-0.10) | <0.01** | | | 4.49 (1.96-10.28) | <0.01** | 3.58 (1.57-8.15) | <0.01** |
| **MD Gender** | | | | | | | | |
| Female | Ref | Ref | Ref | Ref | Ref | Ref | Ref | Ref |
| Male | 1.06 (0.94-1.2) | 0.33 | 1.21 (1.03-1.42) | 0.02* | 0.67 (0.54-0.85) | <0.01** | 0.79 (0.61-1.01) | 0.06 |
| **Patient Volume** | 1.00 (0.99-1.01) | 0.30 | 0.99 (0.99-0.99) | <0.01** | 1.01 (1.01-1.01) | 0.02* | 1.00 (0.99-1.01) | 0.89 |
| **Average Patient Age** | | | | | | | | |
| <65 Years | Ref | Ref | Ref | Ref | Ref | Ref | Ref | Ref |
| >65 Years | 0.76 (0.61-0.93) | 0.01* | 0.74 (0.56-0.98) | 0.03* | 0.73 (0.48-1.12) | 0.14 | 0.54 (0.32-0.91) | 0.02* |
| **MD Clinical Experience (Years)** | | | | | | | | |
| <10 | Ref | Ref | Ref | Ref | Ref | Ref | Ref | Ref |
| 10-19 | 0.99 (0.72-1.36) | 0.96 | 0.85 (0.56-1.28) | 0.42 | 1.67 (0.84-3.35) | 0.14 | 1.27 (0.60-2.69) | 0.53 |
| 20-29 | 1.19 (0.86-1.64) | 0.29 | 1.10 (0.72-1.67) | 0.65 | 1.93 (0.96-3.87) | 0.06 | 1.69 (0.79-3.59) | 0.16 |
| >29 | 1.07 (0.77-1.49) | 0.67 | 0.87 (0.57-1.34) | 0.53 | 2.31 (1.14-4.69) | 0.02* | 2.03 (0.94-4.36) | 0.06 |
| **Surgical Specialty** | | | | | | | | |
| General Surgeon | Ref | Ref | Ref | Ref | Ref | Ref | Ref | Ref |
| Surgical Oncologist | 1.04 (0.87-1.24) | 0.69 | 1.03 (0.81-1.31) | 0.79 | 1.18 (0.86-1.63) | 0.30 | 1.06 (0.74-1.52) | 0.73 |
| **ODX RS Classification** | | | | | | | | |
| Low | | | | | Ref | Ref | Ref | Ref |
| Intermediate | | | | | 7.92 (5.99-10.46) | <0.01** | 8.04 (5.77-11.20) | <0.01** |
| High | | | | | 132.89 (59.4-297.31) | <0.01** | 99.98 (54.92-181.99) | <0.01** |

* significant at the 0.05 level

** significant at the 0.01 level

† ODX eligible patients are defined as stage 1 or 2, LN negative, and HR+/HER2-

**Table S2** Multivariable regression odds ratios for receiving ODX in eligible patients†.

| Variable | Odds Ratio (95% CI) | P-Value |
|---|---|---|
| **Year of Diagnosis** | | |
| 2010 | Ref | Ref |
| 2011 | 1.18 (0.82-1.70) | 0.36 |
| 2012 | 1.00 (0.69-1.45) | 0.98 |
| 2013 | 0.99 (0.69-1.43) | 0.98 |
| 2014 | 1.28 (0.90-1.82) | 0.17 |
| 2015 | 1.21 (0.85-1.72) | 0.28 |
| 2016 | 1.26 (0.87-1.82) | 0.22 |
| **Patient Age at Diagnosis (Years)** | | |
| <50 | Ref | Ref |
| 50-59 | 0.88 (0.67-1.16) | 0.36 |
| 60-69 | 0.68 (0.52-0.90) | <0.01** |
| >69 | 0.26 (0.18-0.37) | <0.01** |
| **Marital Status** | | |
| Single, Divorced, Widowed | Ref | Ref |
| Married | 1.43 (1.18-1.74) | <0.01** |
| Unknown | 1.01 (0.57-1.79) | 0.98 |
| **Grade** | | |
| I | Ref | Ref |
| II | 1.90 (1.56-2.32) | <0.01** |
| III/IV | 1.54 (1.16-2.05) | <0.01** |
| Unknown | 0.30 (0.09-0.94) | 0.04* |
| **Tumor Size (mm)** | | |
| 0.1-19 | Ref | Ref |
| 20-39 | 4.14 (2.69-6.36) | <0.01** |
| >40 | 1.58 (0.81-3.10) | 0.17 |
| Unknown | 0.42 (0.07-2.46) | 0.33 |
| **Clinical Stage** | | |
| 1 | Ref | Ref |
| 2 | 0.70 (0.44-1.12) | 0.13 |
| **MD Clinical Experience (Years)** | | |
| <10 | Ref | Ref |
| 10-19 | 0.69 (0.42-1.14) | 0.14 |
| 20-29 | 0.80 (0.47-1.36) | 0.40 |
| >29 | 0.70 (0.41-1.22) | 0.20 |
| **MD Gender** | | |
| Female | Ref | Ref |
| Male | 1.01 (0.76-1.34) | 0.95 |
| **Patient Volume** | 0.99 (0.98-1.00) | 0.05 |
| **Average Patient Age** | | |
| <65 Years | Ref | Ref |
| >65 Years | 0.74 (0.46-1.20) | 0.21 |
| **Surgical Specialty** | | |
| General Surgeon | Ref | Ref |
| Surgical Oncologist | 0.78 (0.58-1.04) | 0.08 |

* significant at the 0.05 level
** significant at the 0.01 level
† ODX eligible patients are defined as stage 1 or 2, LN negative, and HR+/HER2-

**Table S3** Multivariable regression odds ratios for receiving chemotherapy following ODX.

| Variable | Odds Ratio (95% CI) | P-Value |
|---|---|---|
| **Year of Diagnosis** | | |
| 2010 | Ref | Ref |
| 2011 | 0.99 (0.51-1.93) | 0.98 |
| 2012 | 0.94 (0.47-1.86) | 0.85 |
| 2013 | 0.85 (0.43-1.66) | 0.62 |
| 2014 | 0.56 (0.28-1.09) | 0.08 |
| 2015 | 0.69 (0.35-1.35) | 0.27 |
| 2016 | 0.48 (0.24-0.99) | 0.04* |
| **Patient Age at Diagnosis (Years)** | | |
| <50 | Ref | Ref |
| 50-59 | 0.76 (0.48-1.18) | 0.21 |
| 60-69 | 0.38 (0.24-0.63) | <0.01** |
| >69 | 0.17 (0.07-0.38) | <0.01** |
| **Grade** | | |
| I | Ref | Ref |
| II | 1.52 (0.95-2.41) | 0.07 |
| III/IV | 4.15 (2.42-7.13) | <0.01** |
| Unknown | 0.00 (0.00-Inf) | 0.99 |
| **LN Status** | | |
| Negative | Ref | Ref |
| Positive | 3.17 (1.95-5.15) | <0.01** |
| Unknown | 0.65 (0.16-2.59) | 0.53 |
| **Tumor Size (mm)** | | |
| 0.1-19 | Ref | Ref |
| 20-39 | 1.30 (0.75-2.24) | 0.34 |
| >40 | 4.60 (1.95-10.83) | <0.01** |
| Unknown | 0.79 (0.12-5.44) | 0.81 |
| **Clinical Stage** | | |
| 1 | Ref | Ref |
| 2 | 1.79 (1.01-3.21) | 0.05* |
| 3 / 4 | 2.99 (0.83-10.72) | 0.09 |
| **MD Clinical Experience (Years)** | | |
| <10 | Ref | Ref |
| 10-19 | 1.43 (0.51-3.98) | 0.48 |
| 20-29 | 3.12 (1.11-8.79) | 0.03* |
| >29 | 3.66 (1.26-10.58) | 0.02* |
| **MD Gender** | | |
| Female | Ref | Ref |
| Male | 0.61 (0.40-0.92) | 0.02* |
| **Patient Volume** | 1.00 (0.99-1.02) | 0.89 |
| **Average Patient Age** | | |
| <65 Years | Ref | Ref |
| >65 Years | 0.46 (0.20-1.10) | 0.07 |
| **ODX RS Classification** | | |
| Low | Ref | Ref |
| Intermediate | 12.41 (8.28-18.60) | <0.01** |
| High | 215.90 (100.47-463.93) | <0.01** |

* significant at the 0.05 level
** significant at the 0.01 level

**Table S4** Multivariable regression odds ratios for refusing chemotherapy.

| Variable | Odds Ratio (95% CI) | P-Value |
|---|---|---|
| **Year of Diagnosis** | | |
| 2010 | Ref | Ref |
| 2011 | 1.08 (0.39-2.96) | 0.89 |
| 2012 | 0.66 (0.21-2.09) | 0.48 |
| 2013 | 0.85 (0.28-2.56) | 0.77 |
| 2014 | 2.01 (0.75-5.34) | 0.16 |
| 2015 | 0.58 (0.18-1.88) | 0.36 |
| 2016 | 1.55 (0.52-4.65) | 0.42 |
| **Patient Age at Diagnosis (Years)** | | |
| <50 | Ref | Ref |
| 50-59 | 1.14 (0.54-2.43) | 0.72 |
| 60-69 | 1.92 (0.85-4.31) | 0.11 |
| >69 | 5.62 (1.72-18.39) | <0.01** |
| **Grade** | | |
| I | Ref | Ref |
| II | 1.14 (0.55-2.35) | 0.72 |
| III/IV | 0.43 (0.18-1.03) | 0.05 |
| Unknown | 88980419.30 (0.00-Inf) | 0.99 |
| **LN Status** | | |
| Negative | Ref | Ref |
| Positive | 0.62 (0.30-1.28) | 0.19 |
| Unknown | 1.90 (0.33-10.85) | 0.46 |
| **Tumor Size (mm)** | | |
| 0.1-19 | Ref | Ref |
| 20-39 | 1.13 (0.50-2.58) | 0.76 |
| >40 | 0.38 (0.10-1.40) | 0.14 |
| Unknown | 2.93 (0.17-50.04) | 0.45 |
| **Clinical Stage** | | |
| 1 | Ref | Ref |
| 2 | 0.59 (0.25-1.39) | 0.22 |
| 3 / 4 | 0.52 (0.08-3.29) | 0.48 |
| **MD Clinical Experience (Years)** | | |
| <10 | Ref | Ref |
| 10-19 | 0.79 (0.11-5.50) | 0.80 |
| 20-29 | 0.61 (0.09-4.25) | 0.61 |
| >29 | 0.53 (0.07-3.76) | 0.52 |
| **MD Gender** | | |
| Female | Ref | Ref |
| Male | 0.83 (0.43-1.59) | 0.56 |
| **Patient Volume** | 1.02 (1.01-1.04) | 0.04* |
| **Average Patient Age** | | |
| <65 Years | Ref | Ref |
| >65 Years | 2.57 (0.72-9.20) | 0.14 |
| **ODX RS Classification** | | |
| Low | Ref | Ref |
| Intermediate | 0.30 (0.15-0.60) | <0.01** |
| High | 0.04 (0.01-0.13) | <0.01** |

* significant at the 0.05 level
** significant at the 0.01 level
